# Establishing a theoretical foundation for measuring global health security: a scoping review

**DOI:** 10.1186/s12889-019-7216-0

**Published:** 2019-07-17

**Authors:** Sanjana J. Ravi, Diane Meyer, Elizabeth Cameron, Michelle Nalabandian, Beenish Pervaiz, Jennifer B. Nuzzo

**Affiliations:** 1 The Johns Hopkins Center for Health Security, 621 East Pratt Street, Pier IV Building, Suite 210, Baltimore, MD 21201 USA; 2 Nuclear Threat Initiative, 1776 Eye Street NW, Suite 600, Washington, DC 20006 USA; 3 Watson Institute for International & Public Affairs, Brown University, 111 Thayer Street, Suite 215, Box 1970, Providence, RI 02912 USA

**Keywords:** Global health security, Public health, Assessment, Measurement

## Abstract

**Background:**

Since the 2014–2016 West Africa Ebola epidemic, the concept of measuring health security capacity has become increasingly important within the broader context of health systems-strengthening, enhancing responses to public health emergencies, and reducing global catastrophic biological risks. Efforts to regularly and sustainably track the evolution of health security capabilities and capacities over time – while also accounting for political, social, and environmental risks – could help countries progress toward eliminating sources of health insecurity. We sought to aggregate evidence-based principles that capture a country’s baseline public health and healthcare capabilities, its health security system performance before and during infectious disease crises, and its broader social, political, security, and ecological risk environments.

**Methods:**

We conducted a scoping review of English-language scholarly and gray literature to identify evidence- and practice-based indicators and proxies for measuring health security at the country level over time. We then used a qualitative coding framework to identify recurrent themes in the literature and synthesize foundational principles for measuring global health security. Documents reviewed included English-language literature published after 2001 until the end of the research period—September 2017—to ensure relevance to the current global health security landscape; literature examining acute infectious disease threats with potential for transnational spread; and literature addressing global health security efforts at the country level.

**Results:**

We synthesized four foundational principles for measuring global health security: measurement requires assessment of existing capacities, as well as efforts to build core public health, healthcare, and biosecurity capabilities; assessments of national programs and efforts to mitigate a critical subset of priority threats could inform efforts to generate useful metrics for global health security; there are measurable enabling factors facilitating health security-strengthening efforts; and finally, measurement requires consideration of social, political, and ecological risk environments.

**Conclusion:**

The themes identified in this review could inform efforts to systematically assess the impacts and effectiveness of activities undertaken to strengthen global health security.

**Electronic supplementary material:**

The online version of this article (10.1186/s12889-019-7216-0) contains supplementary material, which is available to authorized users.

## Introduction

“Global health security” refers to prevention, detection, and response to naturally emerging, accidental, and deliberate biological threats [1]. Since the 2014 West Africa Ebola epidemic, the concept of health security has become increasingly important within the broader context of health systems-strengthening, enhancing responses to public health emergencies, and global catastrophic biological risks [2]. In this vein, the World Health Organization’s (WHO) Ebola Interim Assessment Panel, the International Working Group on Financing Preparedness, Chatham House, Harvard University’s Global Health Institute, the National Academy of Medicine, and the World Bank Group have issued calls to improve monitoring and measurement efforts around global health security [1, 3–5].

The WHO’s Joint External Evaluation (JEE) tool partially addresses this need by articulating country-level capacities required to mitigate infectious disease threats; the JEE also establishes a scoring system for quantifying progress made toward meeting benchmarks specified in the International Health Regulations (IHR). However, the JEE process is voluntary and relies on an in-country assessment and in-kind contributions of personnel who conduct the evaluation. While this process remains vital, additional universal approaches to measuring baseline, country-level health security are needed. Efforts to regularly and sustainably track and reproducibly compare the evolution of health security capabilities and capacities over time – while also accounting for political, social, security, and environmental risks – could help countries progress toward eliminating sources of health insecurity.

To inform ongoing efforts to strengthen health systems and establish new mechanisms for monitoring health security -- and as a preliminary step in an ongoing project to develop a Global Health Security Index -- we performed a scoping literature review to articulate foundational principles for measuring global health security. Our objective was to identify evidence-based principles that not only capture health security capabilities before and during infectious disease crises, but also a country’s baseline public health and healthcare capacities and its broader social, environmental, and political risk environments. Themes that emerged from the literature informed our selection of indicators and sub-indicators that could help conceptualize and quantify health security capacities at the country level. In this paper, we summarize the themes we identified in the literature and also offer suggestions for improving future efforts to measure country-level health security.

## Methods

**Fig. 1:**
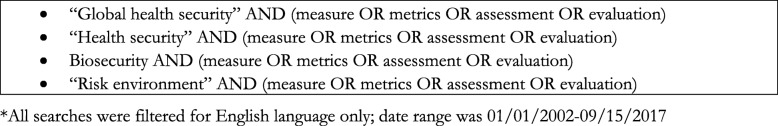
Search Terms Search Terms

We conducted a scoping review of the biomedical and social science scholarly literature, as well as the gray literature. We searched PubMed, Web of Science, and OAIster using the search terms and search limits outlined in Fig. 1. Because this review extracted data from secondary sources and did not involve human subjects research, ethical approval was not required.

Documents eligible for review included only English-language literature published after 2001—to ensure relevance to the current global health security landscape—until the end of the research period (September 2017). Sources were selected if they examined acute infectious disease threats with potential for transnational spread and addressed health security-strengthening efforts at the country level. Documents were excluded if they addressed health security at subnational levels (e.g. county-, district, and/or province-level); biological threats without national or international consequences; or plant, animal, or marine infectious disease threats without known implications for human health.

After exclusion of duplicate titles (i.e., those titles that came up in more than one search), all titles were reviewed by one researcher for relevancy using the above inclusion and exclusion criteria. For those included articles, the abstracts were then reviewed to determine relevancy. All articles deemed relevant were then read in its entirety by a researcher to identify recurrent themes and proxies for measuring global health security. Using NVivo software and a qualitative coding framework developed from a priori themes derived from the JEE and previous global health security research, we coded the documents iteratively, adding new codes to the framework as we identified additional global health security themes and indicators. From the coding process, we synthesized foundational principles for measuring global health security.

## Availability of data and materials

The qualitative coding framework containing all of the themes we identified, and a full list of documents reviewed are provided in Additional file 1 and Additional file 2, respectively.

## Results

**Fig.2:**
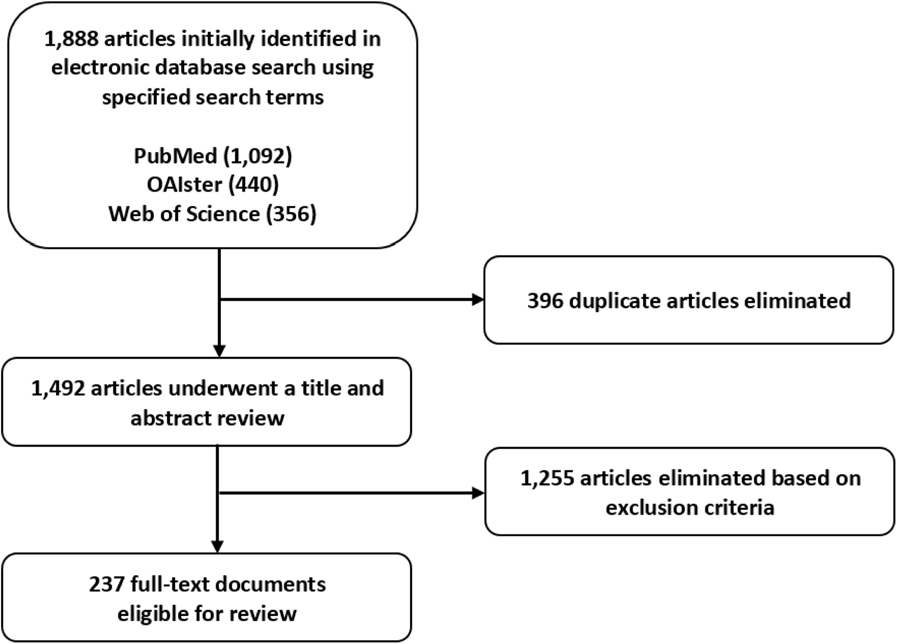
Study Selection

Our search initially yielded 1092 articles from PubMed, 440 articles from OAIster, and 356 articles from Web of Science (1888 total). We eliminated 396 duplicate documents, and, using the aforementioned criteria, eliminated another 1255 documents that were deemed irrelevant upon review of their titles and abstracts, producing a final set of 237 documents which were subsequently coded using NVivo 11 Pro qualitative software (see Fig. 2).

Following are major thematic findings synthesized from our review of the literature, which could serve as foundational principles for measuring global health security. While these thematic findings were derived from our analysis of all 237 documents, we have cited only the documents that we deemed to be most illustrative of said themes within the body of this review. For reference, Additional fil 1 contains a full list of themes, matched with all of the corresponding documents from which they were extracted.

### Measuring global health security requires analysis of existing prevention, detection, and response capacities, as well as efforts to build core public health, healthcare, and biosecurity capacities

Our review broadly affirmed the importance of assessing baseline country capacities for preventing, detecting, and responding to infectious disease threats, an approach widely adopted by existing assessment tools, including the JEE. We identified themes targeting three broad areas: performance of critical health security systems, biosafety and biosecurity, and public health preparedness. With respect to measuring system performance, the literature underscored the importance of assessing biosurveillance systems, emergency response systems, and public health laboratories [6–8]. Specific biosurveillance system capacities and capabilities include the presence of formal programs for monitoring influenza, foodborne pathogens, and wildlife; robust reporting mechanisms; and indicator-based, sentinel surveillance and early warning systems for outbreak detection [9, 10]. We found few descriptions of the operational capabilities required for emergency response; those identified include the ability to coordinate communication between emergency response partners, healthcare surge capacity, the presence of business continuity plans, and sustaining essential services during a crisis [11]. Many of the laboratory system performance themes we identified – including, but not limited to, the presence of national reference laboratories, the quality of diagnostic capacities for priority diseases, protocols for shipping hazardous specimens, and the presence of accreditation and biosafety policies – originated from the IHR Core Capacity Monitoring Framework (2013) [16].

Our review also highlighted the interdependence between global health security, biosecurity, and biosafety. As such, we sought to determine how best to capture the performance of national biosecurity and biosafety mechanisms [12–14]. Given that the definition of “biosecurity” often varies between countries, some of the themes we identified were similarly divergent [15].

Despite some differences in definition, the literature broadly affirmed that robust biosecurity and biosafety mechanisms are critical components of global health security. National laws, regulations, policies, and protocols for enforcing biosecurity and biosafety standards were broadly cited as important components of biosecurity and biosafety at the country level [16–18]. Additionally, the literature consistently underscored the importance of oversight and governance, particularly in the context of reducing risks in the life sciences: national select agent programs, institutional biosafety committees and other deliberative oversight bodies, codes of conduct and ethics, and educational initiatives for scientists and policymakers emerged as important features of robust oversight and governance mechanisms [18–20].

The literature also underscored the essential role of public health preparedness capacities in enhancing health security. Capacities for medical countermeasure development, deployment, and stockpiling – particularly of vaccines – were widely cited as important indicators of health security [21]. Other sources highlighted the importance of access to nonmedical countermeasures (e.g. personal protective equipment, masks, and respirators) in ensuring robust health sector responses to emergent threats [22]. Notably, though our review elicited some indicators for assessing healthcare delivery during infectious disease crises, few documents examined the roles of healthcare in global health security-strengthening efforts. Those that did addressed infection control in clinical settings; the merits and downsides of isolation and quarantine during severe outbreaks; surge capacities during public health crises and mass-casualty events; and coalition-building as a strategy for enhancing regional healthcare capacities [11, 22–24].

Our review also highlighted the importance of risk assessment, which was widely cited as an important tool for characterizing threats across the spectrum of biological risk [25–27]. The literature also emphasized the importance of measuring risk communication capabilities, given the social and economic costs associated with public anxiety, panic, and unrest that often accompanies health crises [28]. Finally, the themes of workforce availability and training cross-cut nearly every health security capacity identified in our review. Healthcare workforces and public health professionals in particular were singled out as critical frontline defenses against emergent threats [28, 29].

### Assessments of national programs and efforts to mitigate a critical subset of priority threats could serve as useful proxies for measuring global health security

Zoonotic diseases trigger devastating economic losses in the agricultural industry and pose threats to human health, particularly for those working in poultry and swine operations. The literature indicated that infection control and occupational guidelines within these operations – including disinfection, vaccination, and use of personal protective equipment – could reduce the risk of disease transmission [30–32]. Programs and policies within the wild game industry (e.g. safe animal handling and regulation of trade between hunters and market owners) are also instrumental in reducing zoonotic transmission [33]. Disease surveillance among wild and domestic animal populations and collaborative approaches to threat mitigation between the human and animal health sectors were also cited as important safeguards against zoonotic disease outbreaks [33].

Widespread emergence of antimicrobial-resistant (AMR) pathogens has diminished the effectiveness of many first-line drugs. Thus, national standards, policies, and programs promoting antimicrobial stewardship (both in human and animal populations) could help mitigate AMR threats, thereby strengthening health security [31, 34]. Additionally, sentinel testing for drug resistance – including among pervasive infectious diseases such as tuberculosis – and increasing access to the diagnostic services needed to detect these pathogens are critical measures for reducing burdens of AMR pathogens [34].

Mass gatherings present additional health security challenges by amplifying the risk of disease transmission both in host countries and countries of returning attendees. As such, the frequency of mass gatherings, as well as the size, location, duration, and season of gathering, could serve as another indicator for measuring countries’ health security vulnerabilities; for example, outbreaks of bacterial meningitis linked to annual Hajj pilgrimages resulted in global spread in 1987 and 2000 [21, 35]. Implementing mandatory vaccination policies, as done by the Saudi Ministry of Health, could help mitigate disease transmission during mass gatherings [21]. Additionally, adherence to global standards for planning for mass gatherings (such as those developed by the WHO) emerged as another important factor to consider when assessing country-level health security [35]. Lastly, the literature noted that mass gatherings could become targets of deliberate biological attacks. In advance of the 2009 Beijing Olympics, for example, China enacted enhanced emergency preparedness measures, including stockpiling pharmaceuticals. Global health security measurements should ideally capture country-level capacities for implementing such measures against deliberate attacks [36].

In addition to efforts addressing mass gatherings, the literature cited national programs targeting risks associated with biotechnology and the life sciences as similarly important components of global health security [37–40]. The life sciences are a critical tool for advancing health security, but could pose threats in the hands of a malicious actor intending to cause deliberate harm. Policies and programs addressing dual research of concern, DURC (i.e. research that could be misused in a way that endangers the public’s health) could ensure proper oversight of entities working with potentially dangerous pathogens. Keys to successful oversight include cooperation between government, academia, the private sector, and law enforcement [41, 42].

### There are measurable enabling factors that facilitate global health security-strengthening efforts

Historical experiences with infectious disease outbreaks can act as an impetus for biosecurity programs, policies, and funding needed to prevent future crises. For example, the September 2001 terrorist and anthrax attacks in the United States catalyzed increased spending and support for biodefense programs [8]. However, there are notable exceptions wherein prior experiences with catastrophic outbreaks in low- and middle-income countries do not always culminate in full preparedness and response capacities across a country or region. This phenomenon has been most recently illustrated by a major outbreak of Ebola in the Democratic Republic of the Congo and an outbreak of Lassa fever in Nigeria, despite the 2014–16 West Africa Ebola outbreak highlighting critical shortcomings in the region’s health security capacities [43, 44]. Still, examining how past crises subsequently trigger changes in spending and programmatic support could elucidate how country-level health security evolves over time.

Additionally, early disease detection and prevention depends on collaboration and communication between health authorities at local, national, regional, and international levels. International norms and strategies play important roles in promoting international collaboration and communication; the IHR, for example, include a directive for signatories to help build health security capacities in resource-poor countries [35, 45]. Other efforts, such as the One Health Initiative, have highlighted intersections between human, environmental, and animal health and the need for greater coordination between these sectors [10]. Evaluating adherence to established norms and incorporation of new approaches to preventing infectious disease crises could aid in determining a country’s collaborative efforts with international partners, as well as the extent to which its animal, human, and environmental health sectors have aligned to tackle emergent threats.

In addition to norms, laws, policies, and regulations also shape health security approaches and outcomes (and vice-versa), and their presence or absence could further modulate a country’s ability to mitigate infectious disease threats. Anema notes, for instance, that many IHR signatories have met the specified core capacity requirements for establishing national legislation and policy; among these states, those which “centralized and harmonized their public health policies and practices” demonstrated greater capacities for overall IHR compliance [16]. Additionally, geopolitical and economic instability were also found to modulate state vulnerability to health security threats; Linacre, for example, notes that countries with low GDPs and primarily agrarian economies are uniquely vulnerable to the threat of agroterrorism, given its potential to slow economic growth. Poor economies, in turn, could subsequently give rise to social unrest and insurgent activity [15].

In addition to highlighting linkages between law, policy, and global health security, the literature broadly affirmed the value of participation in global multilateral institutions (e.g. the World Health Organization; the Global Fund to Fight AIDS, Tuberculosis and Malaria; GAVI, the Vaccine Alliance; and UNAIDS) and compliance with international agreements aimed at strengthening global health security (e.g. the IHR, the Biological and Toxin Weapons Convention, the Cartagena Protocol on Biosafety) [8, 17, 46, 47]. However, at least one article noted that multilateral health initiatives run the risk of establishing parallel health service delivery systems and financing schemes that could disincentivize efforts to build and strengthen in-country mechanisms for mitigating infectious disease threats [17]. Therefore, metrics for evaluating the global risk environment should ideally assess a given country’s health security capabilities against its reliance on supranational governance structures and non-governmental funding streams.

With respect to local and regional collaboration around global health security, the literature highlighted the importance of engaging civil society and private-sector stakeholders, law enforcement, the intelligence community, academia, and political leaders [41, 42, 48, 49]. The extent to which these non-public health entities could serve as another indicator of the robustness of a country’s collaborative health security efforts. Formulation of national strategic plans that coordinate multisector efforts to prevent infectious disease crises could serve as an additional indicator for country-level health security. For example, the U.S. National Strategy for Countering Biological Threats offers guidance for averting catastrophic biological events that could threaten national security [50]. Additionally, funds and resources offered through national programs – such as those offered through the U.S.’s Hospital Preparedness Program – could further incentivize multisector collaboration [51].

Political leadership and commitment are instrumental in ensuring that health security remains a top priority. The U.S. federal government, for example, has launched global health programs, such as the President’s Emergency Plan for AIDS Relief, the President’s Malaria Initiative, the Global Disease Detection Program of the U.S. Centers for Disease Control and Prevention, and the Emerging Pandemic Threats Program through the United States Agency for International Development. Political support also helps ensure adequate funding for biosecurity programs, such as the U.S. Department of Defense Cooperative Threat Reduction Program Biological Threat Reduction Program and the U.S. Department of State Biosecurity Engagement Program. As such, the federal budget typically includes funds for “both biodefense and non-biodefense goals and applications,” which address a range of public health, healthcare, national security, and international security issues in addition to biosecurity, and improve preparedness and response [52, 51]. Globally, sustained financial investments also facilitate country progress toward meeting IHR benchmarks. Besides the U.S., other countries have made financial commitments to strengthening global health security, including Australia, which recently established an Indo-Pacific Centre for Health Security; Finland, which has assumed a leading role in advancing global health security efforts worldwide; Canada, through the Global Affairs Canada Weapons Threat Reduction Program and Public Health Agency Canada; and the Republic of Korea, made an early pledge of USD$100 million to support the Global Health Security Agenda [53–56].

Finally, legislative frameworks for biosecurity and biosafety may also be useful measures of country-level health security. The U.S. Federal Select Agent Program, for example, defines a set of microorganisms and toxins deemed to carry high risks of deliberate misuse and establishes strict security requirements for facilities working with designated high-priority agents; in 2017, the U.S. also implemented policies addressing oversight of pathogens with pandemic potential and enacted a brief moratorium on federally funded gain-of-function studies involving such pathogens [57, 58]. Similarly, in 2008, Israel passed the Regulation of Research into Biological Disease Agents Law, which calls for an oversight body to monitor research involving select, high-risk pathogens [59].

In addition, any legislative frameworks underpinning research should respect the rights of the individual, including ethical considerations when conducting human subject research, and also when implementing public health policies (i.e. quarantine and monitoring policies). Integration of such ethical considerations into legal frameworks will likely become increasingly important in the context of risks associated with biotechnology and the life sciences [60, 61].

### Measuring global health security requires consideration of the risk environment from which infectious disease threats might emerge

The interplay between built and natural environments, climate, and human and animal activity is a critical determinant of global health security [62–64, 46]. Variable economic and political conditions, regulatory environments, modes of governance, laws, and policies concurrently shape state vulnerability to infectious disease threats. As such, the concept of the “risk environment” (i.e. the socioeconomic, political, regulatory, and ecological factors that could give rise to health insecurity) is a useful paradigm for measuring global health security in the context of a country’s unique baseline conditions. Additionally, geopolitical factors, such as the presence of extremist groups, ongoing conflict, and modes of governance should be considered when identifying sources of health insecurity.

Globalization was widely cited as a driving force behind transnational disease spread. Increasing travel, tourism, and trade were described as important promoters of economic growth, but ones that concomitantly elevate the risk of disease transmission among highly mobile populations connected through increasingly accessible modes of global transit [35]. Efforts to measure global health security might endeavor to capture this tension between protecting public health and ensuring unrestricted commercial activity, such as the presence of health checkpoints at ports of entry, trade embargoes, laws or policies restricting the trade of high-risk products, and safety standards for commercial goods. For example, following a 1986 outbreak of bovine spongiform encephalopathy in the United Kingdom, several countries banned imports of live ruminants and beef originating from the UK [47].

The literature also identified urbanization, changes in land use, and evolving agricultural practices as important components of the health security risk environment, given their roles in modulating disease spread between humans and animals, and creating conditions that could enable emergence of novel zoonoses [33, 65, 66]. The risks posed by increased proximity between human and animal populations manifests in other contexts as well; as noted previously, several documents described mass gatherings as potential catalysts of disease transmission, citing public health risks associated with the Hajj and the Olympic Games [21, 35]. Additionally, Brioudes and Gummow note that certain trade and agricultural practices – including swill feeding, illegal wildlife trading, and introductions of undeclared goods – could further heighten the risk of exposing emerging pathogens to susceptible human or animal populations [65].

## Discussion

Our review highlighted country-level considerations that could inform efforts to monitor global health security, including the presence of existing prevention, detection, and response capacities and capabilities; the presence of national programs, policies, and laws to mitigate various kinds of threats; coordination and communication between health authorities at all levels of government, as well as with other sectors (including public and private); political leadership that supports health security issues; as well as engagement in global multilateral institutions and an understanding of the risk environment.

There was considerable overlap between the themes identified in the literature and priorities outlined in the Global Health Security Agenda (GHSA), IHR, and JEE tool. Generally, the literature affirmed that many of the goals and indicators specified in both are valuable benchmarks for assessing baseline, country-level health security capacities. The prevent-detect-respond paradigm articulated in the GHSA serves as a useful organizing principle for conceptualizing global health security, and the JEE tool puts forth a valuable methodology for measuring both health security capacities and country progress toward IHR targets. The JEE also rightly prioritizes inter-sectoral discussion between agencies of national governments and is intended as a tool to begin the development of a national action plan for health security containing specific milestones to be filled and financed over a specific set of timeframes. Given that the JEE consists of subjectively assigned scores based largely on qualitative observation, the comparisons between countries and over years of the JEE process over time remains in question. Therefore, in addition to the JEE, efforts to measure global health security over time might benefit from focusing on outcome-based metrics that capture a country’s demonstrated prevention, detection, and response capabilities during an infectious disease outbreak. With respect to syndromic surveillance, for example, the JEE considers the size and coverage of a given surveillance system, its electronic reporting capacities, and its methods of data validation; by contrast, Glassman suggests that a country measuring the effectiveness of its syndromic surveillance systems should consider metrics such as rates of disease underreporting or numbers of misidentified cases [67]. Other such metrics for measuring functionality and effectiveness of surveillance systems could include whether a country has an established mechanism for regular sharing of surveillance data between human, animal, and environmental health authorities. While both approaches have merits and limitations, focusing on metrics that assess demonstrated capabilities against desired outcomes could also help countries chart actionable paths toward increased IHR compliance and greater health security.

We found little overlap between the global health security literature – which focuses primarily on acute infectious disease threats – and scholarly work on health system resilience, which encompasses a broader universe of core public health and healthcare assets and functions, such as health workforces, financing, universal health coverage, public risk communication, and integration between the sectors involved in mitigating infectious disease threats. Though our review yielded few tangible metrics for measuring healthcare sector performance, it did underscore the critical roles that healthcare capabilities play in strengthening country-level health security. As demonstrated during the 2014 Ebola outbreak, a healthcare sector’s ability to scale up operations in response to an accelerating threat, diagnose and isolate sick patients, and prevent the spread of infection in clinical settings – all while minimizing disruptions to routine healthcare delivery – are crucial determinants of an outbreak’s broader community impacts. However, current frameworks for assessing global health security – including the JEE – feature few indicators addressing core healthcare capacities and capabilities and their integration into emergency response activities. Given that a country’s infectious disease response capabilities depend on the broader functionality of its health systems, these core public health and healthcare capacities should figure more prominently in efforts to conceptualize and measure global health security. For example, researchers have already sought to identify global and public health core competencies for nursing education; a similar explication of core public health and healthcare functions essential to strengthening health security could enhance efforts to improve infectious disease threat mitigation [68].

Notably, our review also highlighted the importance of the risk environment. Historically, the risk environment framework has been used in the context of drug-related harm reduction activities, HIV prevention, and assessments of global nuclear security [69, 70]. However, many measurement efforts do not account for the full spectrum of social, political, and environmental risks that could give rise to, exacerbate, or mitigate infectious disease crises. Extrapolating the risk environment framework to encompass health security challenges at the population- and system-level underscores the interdependence between built, social, and natural environments, and better captures the potential for disease emergence at the human-animal-ecosystem interface. Furthermore, integrating economic, regulatory, and political considerations into measurements of global health security aligns with observed increases in global travel, migration, and commerce, as well as ongoing changes in land use, climate, and geopolitical stability.

In addition to the conceptual challenges associated with measuring global health security, several practical barriers continue posing technical challenges. Even with a strong theoretical foundation, measurement efforts might still be hindered by limited data availability. Many of the metrics employed in the JEE and other tools – both qualitative and quantitative – are not regularly or systematically collected in a standardized manner. Though alternative metrics could support more conceptually sound methods of measurement, poor data availability would still preclude their adoption and meaningful use. International organizations that routinely collect needed metrics should accelerate efforts to identify high-priority data needs, collect said data, and make them readily accessible to the public; the private sector might play an important role in making greater data availability a reality. Finally, although measuring global health security is a critical step in progressing toward greater IHR compliance, measurement alone cannot improve country-level health security capacities. As such, measurement efforts should be linked to incentives (i.e. funding and programmatic support) that promote capacity-building across all sectors involved in infectious disease mitigation.

Our investigation had a few limitations; namely, that the themes identified are a function of available published scholarship. The majority of the articles reviewed were produced by researchers representing predominantly high-income countries. As such, the literature skewed heavily toward themes (e.g. dual-use research of concern) that may not be top health security priorities for policymakers in lower-income countries with differing health security threat landscapes (e.g. greater risks from naturally emerging zoonoses) as compared to industrialized nations. Additionally, though we attempted to systematically review the gray literature, our search engines did not produce many of the foundational documents published by the WHO, ministries of health, and non-governmental groups in the health security space. Future efforts to measure global health security might benefit from additional, complementary modes of data collection, such as consultations with subject matter experts. This might also be useful in further refining those capacities and capabilities that are necessary in countries with poor healthcare and public health infrastructure. Finally, we acknowledge that there is ongoing debate over the definition of global health security itself, and that our inclusion and exclusion criteria – while reflective of the definition used by the WHO – might not have allowed us to identify literature proposing alternative definitions.

## Conclusions

In light of ongoing international initiatives aiming to enhance global capacities and capabilities for infectious disease preparedness and response, the evidence-based themes identified in this review could inform efforts to systematically assess the impacts and effectiveness of activities undertaken to strengthen global health security.

## Additional files

Additional file 1: Coding Framework Coding Framework. The formal coding framework used to identify metrics in the literature for measuring global health security. The definition of each code is provided, along with the number of sources in which the code was used, and the number of times the code was used across all sources. (DOCX 29 kb)

Additional file 2: GHS Literature Review. Comprehensive List of Literature Reviewed (alphabetical) The full list of gray & scholarly publications identified in our review. (DOCX 35 kb)

## Data Availability

Not applicable. No original data were collected over the course of this study; all documents and articles examined are publicly available secondary sources (see Additional file 1 and Additional file 2.

## Abbreviations

AMR: Antimicrobial resistance
GHSA: Global Health Security Agenda
IHR: International Health Regulations
JEE: Joint External Evaluation
WHO: World Health Organization
