# Supplementary material for: Establishing a theoretical foundation for measuring global health security: a scoping review
Source: BMC Public Health. 2019 Jul 17;19:954. doi: 10.1186/s12889-019-7216-0 (PMC6637489; doi:10.1186/s12889-019-7216-0)
Supplement: Supplementary file 1 — Coding Framework Coding Framework. The formal coding framework used to identify metrics in the literature for measuring global health security. The definition of each code is provided, along with the number of sources in which the code was used, and the number of times the code was used across all sources. (DOCX 29 kb) [file 12889_2019_7216_MOESM1_ESM.docx]

**Appendix A: Coding Framework**

Below is the formal coding framework used to identify metrics in the literature for measuring global health security. The definition of each code is provided, along with the number of sources in which the code was used, and the number of times the code was used across all sources (“Number of References”). The specific sources in which each code was identified are listed in the far-right column.

| **Code** | **Definition** | **Number of Sources** | **Number of References** | **Sources Cited in the Review** |
| --- | --- | --- | --- | --- |
| Advocacy | Formal efforts to promote global health security at the highest levels of political leadership | 3 | 4 | Asturias 2016; Barker 2012; Johnson 2013 |
| Agriculture | Steps to address threats to the agricultural sector | 1 | 1 | Linacre 2005 |
| Antimicrobial Resistance | Formal efforts to mitigate the threat of antimicrobial resistance | 15 | 48 | Agunos 2016; Alikhanova 2014; Boeras 2016; Dargatz 2002; Darr 2016; Eichberg 2015; Heymann 2013; Holden 2002; Katz 2015; Kwik 2003; Lo 2017; Marais 2014; Roffey 2015; Schipp 2016; Sumpradit 2012 |
| Bioeconomy | Steps to strengthen a country’s economy through biotechnology-focused scientific and industrial efforts | 1 | 1 | Gottron 2009 |
| Biosafety & Biosecurity | Implementation of policies, laws, regulations, and/or protocols to increase protection from biological threats | 66 | 240 | Agunos 2016; Ancillotti 2016; Baker 2007; Bhattacharjee 2009; Boisvert 2012; Bork 2007; Budowle 2007; Colf 2016; Colussi 2013; Czarkowski 2008; Devaux 2015; Dickmann 2015; Dubov 2014; Duprex 2015; Engel-Glatter 2014; Fears 2016; Federation of UN Association 2007; Fouchier 2012; Friedman 2010; Garrett 2012; Gaudioso 2004; George Washington University 2014; Gottron 2009; Graham 2008; Grunow 2014; Heikkila 2009; Hennessey 2008; Higgins 2013; Huang 2007; Johnson 2013; Katz 2015; Koblentz 2012; Kwik 2003; Lev 2014; Linacre 2005; Lipsitch 2014; Maher 2012; National Research Council 2010; Ndhine 2016; NSABB 2006; Outhwaite 2006; Pavia 2012; Pearson 2007; Puro 2012; Rager-Zisman 2012; Richmond 2003;  Richmond (2) 2003; Rohde 2013; Salerno 2007; Schipp 2016; Selgelid 2016; Shelby 2015; Shurtleff 2012; Sijnesael 2014; Smith 2017; Sundqvist 2013; Tegnell 2006; Thiberville 2012; Tiongco 2009; Trevan 2012; Uhlenhaut 2013; van Aken 2006; Wenzel 2007 WHO 2010; Zaki 2010; Zuckerman 2005 |
| Biosurveillance | National systems for collecting, integrating, and interpreting data on infectious threats to human health | 51 | 113 | Agunos 2016; Aledort 2007; Andrus 2010; Anema 2012; Artois 2009; Baker 2007; Barboza 2012; Barker 2012; Basundra 2016; Bottoms 2013; Brioudes 2016; Burkle 2003; Devaux 2015; Doggett 2016; Ekmekci 2016; Froeschol 2016; Hamblion 2014; Hitchcock 2007; Honigsbaum 2017; Hsu 2017; Ijaz 2012; Katz 2015; Kraemer 2015; Kuchenmuller 2009; Linacre 2005; Lo 2017; MacIntyre 2015; Mackey 2016; Marangon 2008; Miller 2004; Minarcine 2012; Moore 2015; Oshitani 2005; Paquet 2006; Polansky 2016; Quandelacy 2011; Rao 2010; Riccardo 2014; Riccardo 2016; Richmond 2003; Salerno 2007; Smolinski 2017; Stoto 2014; Taboy 2010; Wamala 2010; Waring 2005; Warren 2010; Warren 2013; Watkins 2006; Wei 2015; Wilson 2010 |
| Border Security | Monitoring of travelers at border checkpoints to identify potential cross-border threats | 5 | 8 | Baker 2007; Bogoch 2015; Hamblion 2014; Ho 2017; Linacre 2005 |
| Civil Society | Civil society sector is well integrated into its emergency response infrastructure | 2 | 4 | Ho 2017; Mackey 2016 |
| Data Management | Robust mechanisms for collecting, integrating, and interpreting data before and during a public health emergency | 2 | 3 | Minarcine 2012; Salerno 2007 |
| Decontamination | Technology to neutralize or remove dangerous pathogens from a person or a given environment. | 2 | 3 | Linacre 2005; Raber 2011 |
| DURC | Implementation of experiments that qualify as dual-use research of concern (DURC). | 22 | 44 | Atlas 2003; Berns 2012; Budowle 2007; Hunter 2012; Ingelsby 2012; Kahn 2012; Keim 2012; Koblentz 2012; Lev 2014; Linacre 2005; Lipsitch 2014; MacIntyre 2015; Maher 2012; National Research Council 2010; Novossiolova 2012; Rager-Zisman 2012; Rappert 2007; Rappert 2014; Selgelid 2016; Trevan 2012; Vogel 2014; Wilinetz 2012 |
| Emergency Operations | Robust infrastructure for coordinating communication and activities between the sectors and stakeholders involved in emergency response efforts. | 12 | 28 | Andrus 2010; Barker 2012; Courtney 2009; Diehl 2016; Franco 2006; Froeschol 2016; Hamblion 2014; Hanfling 2013; Hipper 2015; Ijaz 2012; Katz 2015; Vong 2016 |
| Ethics | Steps (e.g. developed a code of conduct, established a deliberative body) to address ethical issues in the life sciences. | 17 | 31 | Hilhorst 2016; Jacobs 2007; Kahn 2012; Link 2011; MacIntyre 2015; Maher 2012; Minarcine 2012; National Research Council 2010; Novossiolova 2012; Rager-Zisman 2012; Rappert 2007; Rohde 2013; Selgelid 2016; Sijnesael 2014; Straetemans 2007; Uhlenhaut 2013; WHO 2010 |
| Food Safety & Security | Formal efforts to prevent and counter infectious disease threats to the food supply | 10 | 43 | Boisvert 2012; Dagg 2006; Heikkila 2009; Hennessey 2008; Ilbery 2012; Katz 2015; Kuchenmuller 2009; Linacre 2005; Noordhuizen 2013; Rao 2010 |
| Forensics | The technology and scientific expertise to investigate biological accidents and/or potential acts of bioterrorism. | 2 | 2 | Gottron 2009; MacIntyre 2015 |
| Funding | Adequate resources to finance global health security-strengthening efforts. | 19 | 40 | Asturias 2016; Canyon 2015; Epstein 2015; Franco 2009; Franco 2010; Garrett 2012; Gottron 2009; Hennessey 2008; Katz 2015; Levi 2009; Linacre 2005; Mackey 2016; Moore 2015; Murray 2012; Ondoa 2016; Toner 2009; Toner 2017; Vong 2016; Wilinetz 2012 |
| Globalization | Awareness of how travel and the growing interaction between people, animals, and different environments can increase risk for disease emergence | 8 | 8 | Barker 2012; Ilbery 2012; Katz 2015; Koblentz 2012; Lo 2017; Nerlich 2011; Outhwaite 2006; Quandelacy 2011 |
| Governance | Mechanisms to ensure sound decision-making and allocation of resources with respect to global health security-strengthening efforts. | 11 | 16 | Dickmann 2016; Katz 2015; Kraemer 2015; Mackey 2016; Ooms 2016; Rager-Zisman 2012; Rappert 2007; Rappert 2014; Rohde 2013; Uhlenhaut 2013; Zuckerman 2005 |
| Oversight | Mechanisms to ensure appropriate scientific conduct in life sciences research. | 10 | 21 | Gottron 2009; Hunter 2012; Lipsitch 2014; Maher 2012; Outhwaite 2006; Pavia 2012; Rager-Zisman 2012; Rappert 2014; Aken 2006; WHO 2010 |
| Healthcare | Implementation of efforts to ensure that the healthcare sector is prepared to respond to infectious disease crises. | 18 | 37 | Canyon 2015; Courtney 2009; Dembek 2017; Froeschol 2016; Gudason 2008; Hales 2014; Khandaker 2017; Koblentz 2012; Levi 2009; Marais 2014; Ortiz 2016; Roffey 2015; Straetemans 2007; Sumpradit 2012; Thaler 2012; Toner 2009; Vong 2016; Zaza 2016 |
| Infection Control | Implementation of laws, regulations, policies, and/or protocols to reduce the risk of infection in clinical settings. | 4 | 7 | Aldedort 2007; Darr 2016; George Washington University 2014; Katz 2015; |
| Isolation & Quarantine | Implementation of laws, regulations, policies, and/or protocols regarding isolation and quarantine of infected persons. | 6 | 8 | Aledort 2007; Desclaus 2017; Jacobs 2007; Kraemer 2015; Levi 2009; Wenzel 2007 |
| Historical Experiences | Previous experience(s) with infectious disease threats inform a country’s current approach to strengthening global health security. | 19 | 30 | Gottron 2009; Jacobson 2014; Lev 2014; Linacre 2005; Lumpkin 2013; Mackey 2016; Maher 2012; Maye 2012; National Research Council 2010; Parnell 2008; Puro 2012; Rager-Zisman 2012; Rappert 2014; Richmond 2014; Rumiko 2015; Salerno 2007; Toner 2009; Vong 2016; Warren 2010 |
| Incentives | Financial and/or programmatic incentives are offered to promote global health security-strengthening efforts. | 11 | 14 | Gottron 2009; Lev 2014; Linacre 2005; Lipsitch 2014; Minarcine 2012; Noordhuizen 2013; Outhwaite 2006; Rumiko 2015; Toner 2009; Wamala 2010; Yeh 2016 |
| Intelligence | The intelligence community is integrated into efforts to investigate infectious disease events posing national security threats. | 8 | 15 | Barboza 2012; Gottron 2009; Lev 2014; Linacre 2005; MacIntyre 2015; Minarcine 2012; Riccardo 2014; Vogel 2014 |
| Inter-agency, Sector, Discipline | Steps to ensure strong collaboration between the numerous sectors, agencies, and disciplines involved in mitigating infectious disease threats. | 32 | 53 | Ahmed 2009; Artois 2009; Berns 2012; Budowle 2007; Burkle 2003; Canyon 2015; Dickmann 2016; Dubov 2014; Gottron 2009; Hipper 2015; Jacobson 2014; Kakkar 2014; Koblentz 2012; Kuchenmuller 2009; Levi 2009; Link 2011; Lo 2017; Lumpkin 2013; MacIntyre 2015; Marais 2014; Minarcine 2012; Murray 2012; Outhwaite 2006; Roffey 2015; Shelton 2012; Su 2017; Taboy 2010; Toner 2009; Toner 2017; Waring 2005; Wenzel 2007; Zaza 2016 |
| International Collaboration | Steps to build collaborative ties with other countries as well as global or multilateral institutions to address global health security threats. | 27 | 36 | Baker 2007; Barboza 2012; Basundra 2016; Devaux 2015; Diehl 2016; Gottron 2009; Johnson 2015; Katz 2015; Koblentz 2012; Levi 2009; Linacre 2005; Moore 2015; Murray 2012; Ooms 2016; Quandelacy 2011; Rao 2010; Roffey 2015; Rohde 2013; Salerno 2007; Shreve 2016; Smith 2017; Thaler 2012; Toner 2017; van Aken 2006; Vong 2016; Yamada 2014; Zuckerman 2005 |
| Norms & Agreements | Formal norms or international agreements are established with partner countries or institutions relating to global health security. | 16 | 41 | Andrus 2010; Anema 2012; Baker 2007; Bogoch 2015; Canyon 2015; Dickmann 2016; Epstein 2015; George Washington University 2014; Gostin 2016; Hamblion 2014; Hitchcock 2007;  Honigsbaum 2017; Ijaz 2012; Katz 2015; Koblentz 2011; Koblentz 2012 |
| Sample Sharing | Protocols are established for sharing clinical samples with different countries. | 2 | 2 | Barker 2012; Polansky 2016 |
| Laboratories | A national and/or regional network of public health laboratories has been established. | 25 | 62 | Aledort 2007; Anema 2012; Barker 2012; Boeras 2016; George Washington University 2014; Gottron 2009; Grunow 2014; Hamblion 2014; Honigsbaum 2017; Ijaz 2012; Katz 2015; Kok 2015; Murray 2012; Ondoa 2016; Polansky 2016; Sealy 2016; Stoto 2014; Sundqvist 2013; Taboy 2010; Wamala 2010; Waring 2005; Westergaard 2007; Yang 2017; Yeh 2016; Zaki 2010 |
| Legal | Presence of laws governing infectious disease prevention, detection, response, and recovery efforts. | 21 | 33 | Anema 2012; Budowle 2007; CDC Select Agents 2012; Gottron 2009; Johnson 2013; Levi 2009; Lumpkin 2013; MacIntyre 2015; Moore 2015; National Research Council 2010; Ndhine 2016; Ooms 2016; Quandelacy 2011; Rager-Zisman 2012; Rappert 2014; Richmond 2014; Serratosa 2004; Uhlenhaut 2013; Wamala 2010; Westergaard 2007; Wilson 2008 |
| Life Sciences Research | Robust research enterprise in the life sciences. | 5 | 8 | National Research Council 2010; Rappert 2007; Rappert 2014; World Health Organization 2010; Wilinetz 2012 |
| Mass Gatherings | Hosting of (or has recently hosted) an event drawing large crowds from different parts of the world. | 3 | 7 | Abd El Ghany 2016; Ahmed 2009; Riccardo 2016 |
| Medical Countermeasures | Efforts to increase medical countermeasure research and development, and ensure MCM deployment during a crisis. | 25 | 66 | Abd El Ghany 2016; Aledort 2007; Andrus 2010; Asturias 2016; Basundra 2016; Elbe 2015; Gottron 2009; Gronvall 2007; Henderson 2011; Kwik 2003; Levi 2009; Mackey 2016; Marais 2014; Marangon 2007; Marangon 2008; Medina 2016; Ortiz 2016; Rager-Zisman 2012; Rebmann 2015; Rumiko 2015; Serratosa 2004; Straetemans 2007; Wei 2015; Westergaard 2007; Zaza 2016 |
| Political Leadership & Commitment | Formal commitments to strengthening their country’s health security capacities. | 3 | 3 | Lumpkin 2013; Roffey 2015; Sumpradit 2012 |
| Preparedness | Steps to prepare for major infectious disease threats in advance of a crisis | 13 | 20 | Andrus 2010; Barker 2012; Blumenstock 2014; Canyon 2015; Chretien 2016; Courtney 2009; Diehl 2016; Johnson 2013; Murray 2012; Mutsaers 2015; Shelton 2013; Shreve 2016; Vong 2016 |
| Private Sector | Private sector organizations have been integrated into the country’s global health security efforts. | 4 | 6 | Gottron 2009; Hester 2012; Levi 2009; Sun 2014 |
| Public Health | The public health sector has been integrated into the country’s global health security efforts. | 5 | 7 | Henderson 2011; Levi 2009; Stoto 2014; Westergaard 2007; Wieland 2011 |
| Reporting | Mechanisms for reporting surveillance system findings to the appropriate authorities. | 22 | 32 | Andrus 2010; Anema 2012; Baker 2007; Barboza 2012; Barker 2012; Bruschke 2008; Gostin 2016; Gottron 2009; Linacre 2005; Lo 2017; Mackey 2016; Medina 2016; Minarcine 2012; Nerlich 2011; Noordhuizen 2013; Oshitani 2005; Quandelacy 2011; Riccardo 2014; Stoto 2014; Su 2017; Taboy 2010; Waring 2005 |
| Research | Ongoing research efforts examining approaches to disaster management, infectious disease threats, and/or biosecurity. | 5 | 5 | Canyon 2015; Ilbery 2012; Inglesby 2012; Levi 2009; Murray 2012 |
| Risk Assessment | Formal efforts to characterize its unique biological risks. | 31 | 44 | Aledort 2007; Berns 2012; Gottron 2009; Hennessey 2009; Hunter 2012; Lev 2014; Linacre 2005; Link 2011; Lipsitch 2014; MacIntyre 2015; Marangon 2008; Maye 2012; McIntyre 2014; Murray 2012; Noordhuizen 2013; Parnell 2008; Proctor 2008; Raber 2011; Rao 2010; Rappert 2014; Richmond 2003; Rohde 2013; Salerno 2007; Shreve 2016; Toner 2009; van Aken 2006; Vogel 2014; Vong 2016; Waring 2005; Watson 2017; Wieland 2011 |
| Risk Communication | Formal policies for communicating and sharing information with the public during an ongoing emergency. | 24 | 54 | Basundra 2016; Bogoch 2015; Briggs 2011; Dembek 2017; Dickmann 2016; Diehl 2016; Ho 2017; Honigsbaum 2017; Hsu 2017; Kraemer 2015; Levi 2009; Link 2011; Maher 2012; Medina 2016; Murray 2012; Nerlich 2011; Noordhuizen 2013; Paquet 2006; Parnell 2008; Scarcella 2013; Shreve 2016; Swain 2015; Vong 2016; Warner 2012 |
| Risk Environment | The socioeconomic, political, regulatory, and ecological factors that could give rise to health insecurity. | 24 | 45 | Baker 2007; Basundra 2016; Canyon 2015; Chretien 2016; Hales 2014; Honigsbaum 2017; Ilbery 2012; Linacre 2005; Medina 2016; Murray 2012; National Research Council 2010; Nerlich 2011; Quandelacy 2011; Rao 2010; Rappert 2014; Rhodes 2005; Shreve 2016; Teng 2017; Thaler 2012; Waring 2005; Warren 2013; Xu 2014; Yamada 2014; Zhen 2015 |
| Standards | Agreed-upon norms for health security system performance. | 7 | 11 | Gottron 2009; Outhwaite 2006; Quandelacy 2011; Rappert 2014; Taboy 2010; Toner 2009; Yeh 2016 |
| Strategic Planning & Coordination | Various sectors involved in addressing health security jointly plan for responding to emergent threats. | 23 | 32 | Andrus 2010; Briggs 2011; Courtney 2009; Devaux 2015; Ekmekci 2016; Gottron 2009; Koblentz 2012; Levi 2009; Lipsitch 2014; Lumpkin 2013; Medina 2016; Minarcine 2012; Murray 2012; Outhwaite 2006; Su 2017; Taboy 2010; Toner 2009; Vong 2016; Wamala 2010; Westergaard 2007; Yeh 2016; Zaza 2016 |
| Surge Capacity | Health sector is capable of scaling up its operations to respond to an emerging crisis. | 5 | 5 | Gottron 2009; Levi 2009; Medina 2016; Waring 2005; Zaza 2016 |
| Technical Assistance | Technical assistance to partner countries responding to infectious disease crises. | 3 | 3 | Ahmed 2009; Asturias 2016; Taboy 2010 |
| Technology | Integration of novel technologies into its preparedness and response efforts. | 3 | 3 | Raber 2011; Rager-Zisman 2012; Rao 2010 |
| Terrorism & Deliberate Misuse | Vulnerability to the threats of biological terrorism or deliberate misuse of dangerous pathogens. | 9 | 12 | Briggs 2011; Huang 2007; Linacre 2005; Lo 2017; Rager-Zisman 2012; Salerno 2007; Smith 2017; Trevan 2012; Zuckerman 2005 |
| Trade & Commerce | Trade and commercial activities that could facilitate cross-border movement of pathogens. | 3 | 4 | Maye 2012; Outhwaite 2006; Serratosa 2004 |
| Travel | Increasing rates of travel could potentially facilitate cross-border movement of pathogens. | 6 | 7 | Ahmed 2009; Basundra 2016; Heymann 2013; Kraemer 2015; Vong 2016; Warren 2010 |
| Vulnerable Populations | Formal efforts to address the needs of its vulnerable populations. | 3 | 4 | Marais 2014; Medina 2016; Ortiz 2016 |
| Workforce | Formal efforts to strengthen its public health, healthcare, community health, and laboratory workforces. | 29 | 46 | Briggs 2011; Charney 2015; Coetsee 2000; Diehl 2016; Froeschol 2016; Gottron 2009; Hamblion 2014; Huang 2007; Ijaz 2012; Levi 2009; Lumpkin 2013; MacIntyre 2015; Minarcine 2012; Olayinka 2016; Oshitani 2005; Puro 2012; Rappert 2014; Rebmann 2015; Richmond 2003; Richmond 2014; Richmond (2) 2003; Stoto 2014; Sundqvist 2013; Thaler 2012; Uhlenhaut 2013; Vong 2016; Wamala 2010; Wei 2015; Westergaard 2007 |
| Training | Formal efforts to ensure that its workforces are adequately trained in emergency preparedness and response procedures. | 7 | 7 | Gottron 2009; Hipper 2015; Johnson 2013; Johnson 2015; MacIntyre 2015; Minarcine 2012; Su 2017 |
| Zoonotic Disease | Formal efforts to address the threat of zoonotic disease. | 23 | 65 | Abdelwhab 2012; Agunos 2016; Artois 2009; Artois 2011; Brioudes 2016; Bruschke 2008; Cheng 2007; Coetsee 2000; Fasina 2006; Fasina 2007; Graham 2008; Heikkila 2009; Heymann 2013; MacIntyre 2015; Mackey 2016; Majra 2009; Marangon 2008; Murray 2012; Noordhuizen 2013; Rao 2010; Serratosa 2004; Villarreal-Chávez 2003; Wenzel 2007 |
