# Supplementary material for: Establishing a theoretical foundation for measuring global health security: a scoping review
Source: BMC Public Health. 2019 Jul 17;19:954. doi: 10.1186/s12889-019-7216-0 (PMC6637489; doi:10.1186/s12889-019-7216-0)
Supplement: Supplementary file 2 — GHS Literature Review. Comprehensive List of Literature Reviewed (alphabetical) The full list of gray & scholarly publications identified in our review. (DOCX 35 kb) [file 12889_2019_7216_MOESM2_ESM.docx]

**Appendix B: Comprehensive List of Literature Reviewed (alphabetical)**

1. Abd El Ghany M, Sharaf H, Hill-Cawthorne GA. Hajj vaccinations - facts, challenges, and hope. International Journal of Infectious Diseases 2016;47:29-37
2. Abdelwhab EM, Hafez HM. Insight into alternative approaches for Control of avian influenza in poultry, with emphasis on highly pathogenic H5N1. Viruses 2012;4:3179-208.
3. Agunos A, Pierson FW, Lungu B, Dunn PA, Tablante N. Review of nonfoodborne zoonotic and potentially zoonotic poultry diseases. Avian Diseases 2016;60:553-75.
4. Ahmed QA, Barbeschi M, Memish ZA. The quest for public health security at Hajj: The WHO guidelines on communicable disease alert and response during mass gatherings. Travel Medicine and Infectious Disease 2009;7:226-30.
5. Aledort JE, Lurie N, Wasserman J, Bozzette SA. Non-pharmaceutical public health interventions for pandemic influenza: an evaluation of the evidence base. BMC Public Health 2007;7(208).
6. Alikhanova N, Akhundova I, Seyfaddinova M, Mammadbayov E, Mirtskulava V, Rüsch-Gerdes S, et al. First national survey of anti-tuberculosis drug resistance in Azerbaijan and risk factors analysis. Public Health Action 2014;4(2):517-23.
7. American Council for the UNU Millennium Project. Worldwide emerging environmental issues affecting the US military. Washington, DC2007.
8. Ancillotti M, Rerimassie V, Seitz SB, Steurer W. An update of public perceptions of synthetic biology: still undecided? Nanoethics 2016;10:309-25.
9. Andrus J, Aguilera X, Olivia O, Aldighieri S. Global health security and the International Health Regulations. BMC Public Health 2010;10(1).
10. Anema A, Druyts E, Hollmeyer HG, Hardiman MC, Wilson K. Descriptive review and evaluation of the functioning of the International Health Regulations (IHR) Annex 2. AGlobalization and health 2012;8(1).
11. Artois M, Bicout D, Gavier-Widen D, Doctrinal D, Globig A, Munster V, et al. Outbreaks of highly pathogenic avian influenza in Europe: the risks associated with wild birds. Rev Sci Tech 2009;28(1):69=92.
12. Artois M, Blancou J, Dupeyroux O, Gilot-Fromont E. Sustainable control of zoonotic pathogens in wildlife: how to be fair to wild animals? Rev Sci Tech 2011;30(3):733-43.
13. Asturias EJ, Wharton M, Pless R, MacDonald NE, Chen RT, Andrews N, et al. Contributions and challenges for worldwide vaccine safety: The Global Advisory Committee on Vaccine Safety at 15 Years. Vaccine 2016;34(3342-49).
14. Atlas R, Campbell P, Cozzarelli NR, Curfman G, Enquist L, Fink G, et al. Statement on the consideration of biodefence and biosecurity. Nature 2003;421:771.
15. Baker M, Forsyth A. The new International Health Regulations: a revolutionary change in global health security. The New Zealand Medical Journal 2007;120(1267).
16. Barboza P, Vaillant L, Mawudeku A, Nelson N, Hartley D, Madoff L, et al. Evaluation of epidemic intelligence systems integrated in the early alerting and reporting project for the detection of A/H5N1 influenza events. PLOS ONE 2013;8(3):1-9.
17. Barker K. Inﬂuenza preparedness and the bureaucratic reﬂex: Anticipating and generating the 2009 H1N1 event. Health & Place 2012;18:701-09.
18. Basunda S, Hiremath R, Khajuria R, Ghodke S. Zika virus: an emerging public health challenge. Journal of Krishna Institute of Medical Sciences University 2016;5(3):5-12.
19. Bhattacharjee Y. Faulty risk analysis puts biofacility plan in jeopardy. Science 2009;325(5941):661.
20. Blumenstock J, Bakker G, Jarris P. Measuring preparedness: the national health security preparedness index. Journal of Public Health Management and Practice 2014;20(3):361-63.
21. Boeras D, Peeling R, Onyebujoh P, Yahaya A, Gumede-Moeletsi H, Ndihokubwayo J. The WHO AFRO external quality assessment programme (EQAP): linking laboratory networks through EQA programmes. African Journal of Laboratory Medicine 2016;5(2).
22. Bogoch I, Creatore M, Cetron M, Brownstein J, Pesik N, Miniota J, et al. Assessment of the potential for international dissemination of Ebola virus via commercial air travel during the 2014 west African outbreak. Lancet 2014;385(29-35).
23. Boisvert R, Kay D, Turvey C. Macroeconomic costs to large scale disruptions of food production: The case of foot- and-mouth disease in the United States. Economic Modelling 2012;29:1921-30.
24. Bork K, Halkjae-Knudsen V, Hansen J, Heegard E. Biosecurity in Scandinavia. Biosecurity and Bioterrorism: Biodefense Strategy, Practice, and Science 2007;5(1):62-72.
25. Bottoms K, Poljak Z, Friendship R, Deardon R, Alsop J, Dewey C. An assessment of external biosecurity on southern Ontario swine farms and its application to surveillance on a geographic level. The Canadian Journal of Veterinary Research 2013;77:241-53.
26. Briggs C. Communicating biosecurity. Medical Anthropology 2011;30(1):6-29.
27. Brioudes A, Gummow B. Field application of a combined pig and poultry market chain and risk pathway analysis within the Paciﬁc Islands region as a tool for targeted disease surveillance and biosecurity. Preventive Veterinary Medicine 2016;129:13-22.
28. Bruschke C, Vallat B. OIE standards and guidelines related to trade and poultry diseases. 2008.
29. Budowle B, Beaudry J, Barnaby N, Giusti A, Bannan J, Keim P. Role of law enforcement response and microbial forensics in investigation of bioterrorism. Croat Med J 2007;48:437-49.
30. Burkle F. Measures of effectiveness in large-scale bioterrorism events. Prehospital Disaster Medicine 2003;18(3):258-62.
31. Burns K, Casadevall A, Cohen M, Ehrlich S, Enquist L, Fitch J, et al. Adaptations of avian flu virus are a cause for concern. Science 2012;335(660-661).
32. Canyon D, Burkle F, Speare R. Managing community resilience to climate extremes, rapid unsustainable urbanization, emergencies of scarcity, and biodiversity crises by use of a disaster risk reduction bank. Disaster Medicine and Public Health Preparedness 2015;9(6):619-24.
33. Charney R, Rebmann T, Flood R. Hospital employee willingness to work during earthquakes versus pandemics. Journal of Emergency Medicine 2015;49(5):665-74.
34. Cheng V, Lau S, Woo P, Yuen K. Severe acute respiratory syndrome coronavirus as an agent of emerging and reemerging infection. Clinical Microbiology Reviews 2007;20(4):660-94.
35. Coetsee T, Lourens A, Nel M, van der Westhuizen E. Disasters and diseases in Africa: information support for animal and human health. Onderstepoort, South Africa2000.
36. Cohen J. Flu study raises questions about U.S. ban. Science 2015;349(6253):1153.
37. Colf L. Preparing for nontraditional biothreats. Health Security 2016;14(1):7-12.
38. Colussi I. Synthetic biology between challenges and risks: suggestions for a model of governance and a regulatory framework, based on fundamental rights. Rev Derecho Genoma Hum 2013;38:185-214.
39. Courtney B, Toner E, Waldhorn R, Franco C, Rambhia K, Norwood A, et al. Healthcare coalitions: the new foundation for national healthcare preparedness and response for catastrophic health emergencies. Biosecurity and Bioterrorism: Biodefense Strategy, Practice, and Science 2009;7(2):154-63.
40. Czarkowski M. The dilemma of dual use biological research: Polish perspective. Sci Eng Ethics 2010;16(99-110).
41. Dagg P, Butler R, Murray J, Biddle R. Meeting the requirements of importing countries: practice and policy for on-farm approaches to food safety. Rev Sci Tech Off Int Epiz 2006;25(2):685-700.
42. Dar O, Hasan R, Schlundt J, Harbarth S, Caleo G, Dar F, et al. Antimicrobials: access and sustainable eﬀ ectiveness: exploring the evidence base for national and regional policy interventions to combat resistance. Lancet 2016;387:285-95.
43. Dargatz D, Garry F, Traub-Dargatz J. An introduction to biosecurity of cattle operations. Ven Clin Food Anim 2002;18:1-5.
44. Dembek Z, Mothershead J, Chekol T, Myers D, Meris R, Meranus D, et al. Operational perspective of lessons learned from the Ebola crisis. Military Medicine 2017;182:1507-13.
45. Desclaux A, Badji D, Ndione A, Sow K. Accepted monitoring or endured quarantine? Ebola contacts' perceptions in Senegal. Social Science & Medicine 2017;178:38-45.
46. Devaux C. The hidden face of academic researches on classiﬁed highly pathogenic microorganisms. Infection, Genetics and Evolution 2015;29:26-34.
47. Dickmann P, Apfel F, Biedenkopf N, Eickmann M, Becker S. Marburg Biosafety and Biosecurity Scale (MBBS): a framework for risk assessment and risk communication. Health Security 2015;13(2):88-95.
48. Dickmann P, Bhatiasevi A, Chaib F, Baggio O, Banluta B, Hollenweger L, et al. Biological risks to public health: lessons from an international conference to inform the development of national risk communication strategies. Health Security 2016;14(6):433-40.
49. Diehl G, Bradstreet N, Monahan F. The Department of Defense at the forefront of a global health emergency response: lessons learned from the Ebola outbreak. Health Security 2016;14(5):366-74.
50. Doggett N, Mukundan H, Lefkowitz E, Slezak T, Chain P, Morse S, et al. Culture-independent diagnostics for health security. Health Security 2016;14(3):122-42.
51. Dubov A. The concept of governance in dual-use research. Med Health Care and Philos 2014;17:447-57.
52. Dudley J. Public health and epidemiological considerations for avian influenza risk mapping and risk assessment. Ecology and Society 2008;13(2).
53. Duprex W, Fouchier R, Imperiale M, Lipsitch M, Relman D. Gain-of-function experiments: time for a real debate. Nature 2015;13:58-64.
54. Eichberg M. Public funding of clinical-stage antibiotic development in the United States and European Union. Health Security 2015;13(3):156-65.
55. Ekmecki P. An assessment of coherence between early warning and response systems and serious cross-border health threats in the European Union and Turkey. Disaster Medicine and Public Health Preparedness 2016;10(6):883-92.
56. Elbe S, Roemer-Mahler A, Long C. Medical countermeasures for national security: a new government role in the pharmaceuticalization of society. Scocial Science & Medicine 2015;131:263-71.
57. Engel-Glatter S. Dual-use research and the H5N1 bird flu: is restricting publication the solution to biosecurity issues? Science and Public Policy 2014;41:370-83.
58. Epstein G. Biosecurity 2011: not a year to change minds. Bulletin of the Atomic Scientists 2011;68(1):29-38.
59. Fasina F, Joannis T, Abolnik C, McCrindle C, Bisschop S. Community measures: a key to highly pathogenic avian influenza control in developing nations. Intern J Appl Res Vet Med 2006;4(3):249-54.
60. Fasina F, Meseko A, Joannis T, Shittu A, Ularamu H, Egbuji N, et al. Control versus no control: options for avian influenza H5N1 in Nigeria. Zoonoses and Public Health 2007;54:173-6.
61. Fears R, terMeulen V. European academies advise on gain-of-function studies in influenza virus research. Journal of Virology 2016;90(5):2162-4.
62. Fischer J. IHR (2005) compliance: laboratory capacities and biological risks. Washington, DC2014.
63. Fouchier R, Garcia-Sastre A, Kawaoka Y. The pause on avian H5N1 influenza virus transmission research should be ended. mBio 2012;3(5):1-2.
64. Franco C. Billions for Biodefense: Federal Agency Biodefense Funding, FY2009-FY2010. Biosecurity and Bioterrorism: Biodefense Strategy, Practice, and Science 2009;7(3):291-309.
65. Franco C, Sell T. Federal Agency Biodefense Funding: FY2010-FY2011. Biosecurity and Bioterrorism: Biodefense Strategy, Practice, and Science 2010;8(2):129-49.
66. Franco C, Toner E, Waldhorn R, O'Toole BMT, Inglesby T. Systemic collapse: medical care in the aftermath of Hurricane Katrina. Biosecurity and Bioterrorism: Biodefense Strategy, Practice, and Science 2006;4(2):135-46.
67. Friedman D, Rager-Zisman B, Bibi E, Keynan A. The bioterrorism threat and dual-use biotechnological research: an Israeli Perspective. Sci Eng Ethics 2010;16:85-97.
68. Froeschl G, Ntinginya N, Sangare A, Lawala P, Mangu C, Dobler G, et al. Integrating local, national, and international stakeholders in outbreak preparedness in developing countries: conclusions from a conference in Mbeya, Tanzania. Health Security 2016;14(1):29-34.
69. Garrett L. Reporting on biosecurity from America to Zaire. Bulletin of the Atomic Scientists 2012;68(1):1-9.
70. Gaudioso J, Salerno R. Biosecurity and research: minimizing adverse impacts. Science 2004;304:687-.
71. Gostin L, Katz R. The International Health Regulations: the governing framework for global health security. Milbank Quarterly 2016;94:264-313.
72. Gottron F, Shea D. Oversight of high-containment biological laboratories: issues for Congress. Washington, DC2009.
73. Graham J, Leibler J, Price L, Otte J, Pfeiffer D, Tiensin T, et al. The animal-human interface and infectious disease in industrial food animal production: rethinking biosecurity and biocontainment. Public Health Reports 2008;123:282-99.
74. Gronvall G, Trent D, Borio L, Brey R, Nagao L. The FDA animal efficacy rule and biodefense. Nature Biotechnology 2007;25(10):1084-7
75. Grunow R, Ippolito G, Jacob D, Sauer U, Rohleder A, Caro AD, et al. Benefits of a European project on diagnostics of highly pathogenic agents and assessment of potential "dual use" issues. Frontiers in Public Health 2014;2(199):1-11.
76. Gudnason T. An interactive central database of vaccinations in Iceland. Eurosurveillance 2008;13(1):1.
77. Hamblion E, Salter M, Jones J. Achieving compliance with the International Health Regulations by overseas territories of the United Kingdom of Great Britain and Northern Ireland. Bulletin of the World Health Organization 2014;92:836-43.
78. Hanfling D, Bouri N. Foreign medical teams: what role can they play in response to a catastrophic disaster in the US? Disaster Medicine and Public Health Preparedness 2013;7(6):555-62.
79. Heikkilä J. Economics of biosecurity across levels of decision-making: a review. Agron Sustain Dev 2011;31:119-38.
80. Henderson D. The eradication of smallpox -- an overview of the past, present, and future. Vaccine 2011;295:7-9.
81. Henderson D. Smallpox virus destruction and the implications of a new vaccine. Biosecurity and Bioterrorism: Biodefense Strategy, Practice, and Science 2011;9(2):163-8.
82. Hennessey D. Economic aspects of agricultural and food biosecurity. Biosecurity and Bioterrorism: Biodefense Strategy, Practice, and Science 2008;6(1):66-77.
83. Hennessey M, Lee B, Goldsmith T, Halvorson D, Hueston W, McElroy K, et al. Supporting business continutiy during a highly pathogenic avian influenza outbreak: a collaboration of industry, academia, and government. Avian Diseases 2010;54:387-9.
84. Hester S, Cacho O. The contribution of passive surveillance to invasive species management. Biol Invasions 2017;19(737-48):737-48.
85. Heymann D, Dixon M. Infections at the animal/human interface: shifting the paradigm from emergency response to prevention at source. Current Topics in Microbiology and Immunology 2013;366:207-15.
86. Higgins J, Weaver P, Fitch J, Johnson B, Pearl R. Implementation of a personnel reliability program as a facilitator of biosafety and biosecurity culture in BSL-3 and BSL-4 laboratories. Biosecurity and Bioterrorism: Biodefense Strategy, Practice, and Science 2013;11(2):130-7.
87. Hilhorst D, Hodgson L, Jansen B, Mena R. Security guidelines for field research in complex, remote and hazardous places. Rotterdam, the Netherlands: International Institute of Social Studies; 2016.
88. Hipper T, Orr A, Chernak E. Are human service agencies ready for disasters? Findings from a mixed-methods needs assessment and planning project. Health Security 2015;13(2):106-14.
89. Hitchcock P, Chamberlain A, Wagoner MV, Inglesby T, O'Toole T. Challenges to global surveillance and response to infectious disease outbreaks of international importance. Biosecurity and Bioterrorism: Biodefense Strategy, Practice, and Science 2007;5(3):206-27.
90. Ho L, Tsai Y, Lee W, Liao S, Wu L, Wu Y. Taiwan's travel and border health measures in response to Zika. Health Security 2017;15(2):185-91.
91. Holden P, Carr J, Honeyman M, Kliebenstein J, McKean J, Harmon J, et al. Minimizing the Use of Antibiotics in Pork Production. Ames, Iowa: Iowa Pork Industry Center; 2002.
92. Honigsbaum M. Between securitisation and neglect: managing Ebola at the borders of global health. Med Hist 2017;61(2):270-94.
93. Hsu Y, Chen Y, Wei H, Yang W, Chen Y. Risk and outbreak communication: lessons from Taiwan's experiences in the Post-SARS era. Health Security 2017;15(2):165-69.
94. Huang Y. Managing biosecurity threats in China. Biosecurity and Bioterrorism: Biodefense Strategy, Practice, and Science 2011;9(1):31-40.
95. Hunter P. H5N1 infects the biosecurity debate. EMBO Reports 2012;13(7):604-7.
96. Ijaz K, Kasowski E, Arthur R, Angulo F, Dowell S. International Health Regulations - what gets measured gets done. Emerging Infectious Diseases 2012;18(7):1054-7.
97. Ilbery B. Interrogating food security and infectious animal and plant diseases: a critical introduction. The Geographical Journal 2012;178(4):308-12.
98. Inglesby T. Engineered H5N1: a rare time for restraint in science. Annals of Internal Medicine 2012;156:460-2.
99. Jacobs L. Rights and quarantine during the SARS global health crisis: differentiated legal consciousness in Hong Kong, Shanghai, and Toronto. Law & Society Review 2007;41(3):511-52.
100. Jacobson E, Inglesby T, Khan A, Rajotte J, Burhans R, Slemp C, et al. Design of the National Health Security Preparedness Index. Biosecurity and Bioterrorism: Biodefense Strategy, Practice, and Science 2014;12(3):122-31.
101. Johnson A, Akhundova G, Aliyeva S, Strelow L. Implementation and evaluation of a training program as part of the cooperative biological engagement program in Azerbaijan. Frontiers in Public Health 2015;3:1-9.
102. Jonsson C, Cole K, Roy C, Perlin D, Byrne G. Challenges and practices in building and implementing biosafety and biosecurity programs to enable basic and translational research with select agents. J Bioterror Biodef 2014;3(15).
103. Jordan H, Dunt D, Hollingsworth B, Firestone S, Burgman M. Costing the morbidity and mortality consequences of zoonoses using health-adjusted life years. Transboundary and Emerging Diseases 2014;63:301-12.
104. Kahn L. Can biosecurity be embedded into the culture of the life sciences? Biosecurity and Bioterrorism: Biodefense Strategy, Practice, and Science 2012;10(2):241-6.
105. Kakkar M, Abbas S, Hossain S. One Health: a perspective from the human health sector. Rev Sci Tech Off Int Epiz 2014;33(2):407-12.
106. Katz R, Sorrell E, Kornbler S, Fischer J. Global Health Security Agenda and the International Health Regulations: Moving Forward. Biosecurity and Bioterrorism: Biodefense Strategy, Practice, and Science 2014;12(5):231-8.
107. Keim P. The NSABB recommendations: rationale, impact, and implications. mBio 2012;3(1):1-2.
108. Khandaker G, Beard F, Dey A, Coulter C, Hendry A, Macartney K. Evaluation of bacille Calmette-Guérin immunisation programs in Australia. Commun Dis Intell Q Rep 2017;41(1):33-48.
109. Koblentz G. From biodefence to biosecurity: the Obama administration’s strategy for countering biological threats. International Affairs 2012;88(1):131-48.
110. Koblentz G, Chevrier M. Modernizing confidence-building measures for the Biological Weapons Convention. Biosecurity and Bioterrorism: Biodefense Strategy, Practice, and Science 2011;9(3):232-8.
111. Kok J, Ng J, Li S, Giannoutsos J, Nayyar V, Iredell J, et al. Evaluation of point-of-care testing in critically unwell patients: comparison with clinical laboratory analysers and applicability to patients with Ebolavirus infection. Pathology 2015;47(5):405-9.
112. Kraemer J, Siedner M, Stoto M. Analyzing variability in ebola related controls applied to returned travelers in the United States. Health Security 2015;13(5):295-306.
113. Kuchenmüller T, Hird S, Stein S, Kramarz P, Nanda A, Havelaar A. Estimating the global burden of foodborne diseases - a collaborative effort. Eurosurveillance 2009;14(18):1-4.
114. Kwik G, Fitzgerald J, Inglesby T, O'Toole T. Biosecurity: responsible stewardship of bioscience in an age of catastrophic terrorism. Biosecurity and Bioterrorism: Biodefense Strategy, Practice, and Science 2003;1(1):27-35.
115. Lev O, Samimian-Darash L. Biosecurity policy in the US: a critical assessment. Frontiers in Public Health 2014;2(110):1-3.
116. Levi J, Inglesby T, Segal L, Vinter S. Pandemic flu preparedness: lessons from the frontlines. Washington, DC: Trust for America's Health; 2009.
117. Linacre N, Koo B, Rosegrant M, Msangi S, Falck-Zepeda J, Gaskell J, et al. Security analysis for agroterrorism: applying the threat, vulnerability, consequence framework to developing countries. Washington, DC: International Food Policy Research Institute; 2005.
118. Link H. Playing God and the intrinsic value of life: moral problems for synthetic biology. Sci Eng Ethics 2013;19:435-48.
119. Lipsitch M, Inglesby T. Moratorium on research intended to create novel potential pandemic pathogens. mBio 2014;5(6):1-6.
120. Lo Y. Implementation of the IHR Joint External Evaluation: Taiwan's experiences. Health Security 2017;15(2):132-6.
121. Lumpkin J, Miller Y, Inglesby T, Links J, Schwartz A, Slemp C, et al. The importance of establishing a National Health Security Preparedness Index. Biosecurity and Bioterrorism: Biodefense Strategy, Practice, and Science 2013;11(1):81-.
122. MacIntyre C. Biopreparedness in the age of genetially engineered pathogens and open access science: an urgent need for a paradigm shift. Military Medicine 2015;180:943-9.
123. Mackey T. The Ebola outbreak: catalyzing a "shift" in global health governance. BMC Infectious Diseases 2016;16(699):1-12.
124. Maher B. The biosecurity oversight. Nature 2012;485:431-4.
125. Majra J, Gur A. Climate change and health: why should India be concerned? Indian Journal of Occupational & Environmental Medicine 2009;13(1):11-6.
126. Marais B, Graham S. Childhood tuberculosis: a roadmap towards zero deaths. Journal of Paediatrics and Child Health 2016;52:258-61.
127. Marangon S, Cecchinato M, Capua I. Use of vaccination in avian influenza control and eradication. Zoonoses and Public Health 2007;55:65-72.
128. Marangon S, Cristalli A, Busani L. Planning and executing a vaccination campaign against avian influenza. Developments in Biologicals 2007;130:99-108.
129. Maye D, Dibden J, Higgins V, Potter C. Governing biosecurity in a neoliberal world: comparative perspectives from Australia and the United Kingdom. Environment and Planning 2012;44:150-68.
130. McIntyre K, Setzkorn C, Hepworth P, Morand S, Morse A, Baylis M. A quantitative prioritisation of human domestic animal pathogens in Europe. PLOS ONE 2014;9(8):1-9.
131. Medina M. Pandemic influenza planning for the mental health security of survivors of mass deaths. In: Masys A, editor. Exploring the Security Landscape: Non-Traditional Security Challenges. Leicester, United Kingdom: Springer; 2016. p. 79-100.
132. Miller M, Roche P, Spencer J, Deeble M. Evaluation of Australia's National Notifiable Disease Surveillance System. Commun Dis Intell Q Rep 2004;28(3):311-23.
133. Minarcine S. Health security intelligence: assessing the nascent public health capability. Department of National Security Affairs. Monterey, CA: Naval Postgraduate School; 2012. p. 85.
134. Moore M, Fisher G, Stevens C. Toward integrated DoD biosurveillance. Washington, DC: The RAND Corporation; 2015.
135. Murray K, Skerratt L, Speare R, Ritchie S, Sout F, Hedlefs R, et al. Cooling off health security hot spots: Getting on top of it down under. Environment International 2012;48:56-64.
136. Mutsaers I. One-health approach as counter-measure against “autoimmune” responses in biosecurity. Social Science & Medicine 2015;129:123-30.
137. National Research Council of the National Academies. Sequence-based classification of select agents. In: Academies TN, editor. Washington, DC. 2010.
138. National Science Advisory Board for Biosecurity. Addressing biosecurity concerns related to the synthesis of select agents. Washington, DC. 2006.
139. Ndhine E, Slotved H, Osoro M, Olsen K, Rugutt M, Wanjohi C, et al. A biosecurity survey in Kenya, November 2014 to February 2015. Health Security 2016;14(4):205-13.
140. Nerlich B, Koteyko N. Crying wolf? Biosecurity and metacommunication in the context of the 2009 swine ﬂu pandemic. Health & Place 2012;18:710-17.
141. Noordhuizen J, Surborg H, Smulders F. On the efficacy of current biosecurity measures at EU borders to prevent the transfer of zoonotic and livestock diseases by travellers. Veterinary Quarterly 2013;33(3):161-71.
142. Novossiolova T, Sture J. Towards the responsible conduct of scientific research: is ethics education enough? Med Confl Surviv 2012;28(1):73-84.
143. Olayinka O, Akpinar-Elci M. Development of occupational health measures for the National Health Security Preparedness Index. Frontiers in Public Health 2016;4(79):1-3.
144. Ondoa P, Datema T, Keita-Sow M, Ndihokubwayo J, Isadore J, Oskam L, et al. A new matrix for scoring the functionality of national laboratory networks in Africa: introducing the LABNET scorecard. African Journal of Laboratory Medicine 2016;5(3).
145. Ooms G, Hammonds R. Global constitutionalism, applied to global health governance: uncovering legitimacy deficits and suggesting remedies. Globalization and Health 2016;12(84).
146. Ortiz J, Perut M, Dumolard L, Wijesinghe P, Jorgensen P, Ropero A, et al. A global review of national inﬂuenza immunization policies: analysis of the 2014 WHO/UNICEF joint reporting form on immunization. Vaccine 2016;34:5400-5.
147. Oshitani H, Ailan L, Roces M, Sian D, Ken C, Kiedrzynski T. Implementing the new International Health Regulations in the Pacific - challenges and opportunities. Pacific Health Surveillance and Response 2005;12(2):135-43.
148. Outhwaite O. Evaluating biosecurity law and regulation in developing countries: case studies from Belize. Department of Law. Greenwich, United Kingdom: University of Greenwich; 2006. p. 514.
149. Paquet C, Coulombier D, Kaiser R, Ciotti M. Epidemic intelligence: a new framework for strengthening disease surveillance in Europe. Eurosurveillance 2006;11(12):212-14.
150. Parnell G, Borio L, Brown G, Banks D, Wilson A. Scientists urge DHS to improve bioterrorism risk assessment. Biosecurity and Bioterrorism: Biodefense Strategy, Practice, and Science 2008;6(4):353-56.
151. Pavia A. Laboratory creation of a highly transmissible H5N1 influenza virus: balancing substantial risks and real benefits. Annals of Internal Medicine 2012;156:463-5.
152. Pearson J. Regulatory constraints for the transport of samples and compliance with the World Organisation for Animal Health (OIE) standards for biosecurity and biocontainment. Dev Biol (Basel) 2007;128:59-68.
153. Polansky L, Outin-Blenman S, Moen A. Improved global capacity for influenza surveillance. Emerging Infectious Diseases 2016;22(6):993-1000.
154. Potter M, Houck O, Miner K, Shoaf K. Data for preparedness metrics: legal, economic, and operational. J Public Health Manag Pract 2013;10(2):22-7.
155. Proctor R. The significance of consequence assessment applied to the risk based approach of homeland security. Center for Homeland Defense and Security. Monterey, California: Naval Postgraduate School; 2008.
156. Puro V, Fusco F, Schilling S, Thomson G, Iaco GD, Broqui P, et al. Biosecurity measures in 48 isolation facilities managing highly infectious diseases. Biosecurity and Bioterrorism: Biodefense Strategy, Practice, and Science 2012;10(2 ):208-14.
157. Quandelacy T, Johns M, Andraghetti R, Hora R, Meynard J, Montgomery J, et al. The role of disease surveillance in achieving IHR compliance by 2012. Biosecurity and Bioterrorism: Biodefense Strategy, Practice, and Science 2011;9(4):408-12.
158. Raber E. The challenge of determining the need for remediation following a wide-area biological release. Biosecurity and Bioterrorism: Biodefense Strategy, Practice, and Science 2011;9(3):257-61.
159. Rager-Zisman B. Ethical and regulatory challenges posed by synthetic biology. Perspectives in Biology and Medicine 2012;55(4):590-607.
160. Rao V. Integrated regional bioengagement framework to combat brucellosis. Prilozi 2010;31(1):191-207.
161. Rappert B. Codes of conduct and biological weapons: an in-process assessment. Biosecurity and Bioterrorism: Biodefense Strategy, Practice, and Science 2007;5(2):145-54.
162. Rappert B. Why has not there been more research of concern? Frontiers in Public Health 2014;2(74):1-14.
163. Rebmann T, Loux T, Swick Z, Dolgin H, Reddick D, Wakefield M. Are US jurisdictions prepared to dispense medical countermeasures through open points of dispensing? Findings from a national study. Health Security 2015;13(2):96-105.
164. Rhodes T, Simic M. Transition and the HIV risk environment. BMJ 2005;331:220-23.
165. Riccardo F, Manso MD, Caporali M, Napoli C, Linge J, Mantica E, et al. Event-based surveillance during EXPO Milan 2015: rationale, tools, procedures, and initial results. Health Security 2016;14(3):161-72.
166. Riccardo F, Shigematsu M, Chow C, McKnight C, Linge J, Doherty B, et al. Interfacing a biosurveillance portal and an international network of institutional analysts to detect biological threats. Biosecurity and Bioterrorism: Biodefense Strategy, Practice, and Science 2014;12(6):325-36.
167. Richmond A, Sobelson R, Cioffi J. Preparedness and emergency response learning centers: supporting the workforce for national health security. J Public Health Manag Pract 2014;20(5):7-16.
168. Richmond J, Hill R, Weyant R, Nesby-O'Dell S, Vinson P. What's hot in animal biosafety? ILAR Journal 2003;44(1):20-7.
169. Richmond J, Nesby-O'Dell S. Biosecurity for animal facilities and associated laboratories. Lab Animal 2003;32(1):32-5.
170. Roffey R, Lindberg A, Molin L, Wikman-Svahn P. A plausible worst-case scenario of increasing multidrug resistance as a tool for assessing societal risks and capabilities in Sweden. Health Security 2015;13(3):174-83.
171. Rohde C, Smith D, Martin D, Fritze D, Stalpers J. Code of conduct of biosecurity for biological resource centres: procedural implementation. International Journal of Systematic and Evolutionary Microbiology 2013;63:2374-82.
172. Salerno R, Hickok L. Strengthening bioterrorism prevention: global biological materials management. Biosecurity and Bioterrorism: Biodefense Strategy, Practice, and Science 2007;5(2):107-16.
173. Scarcella C, Antonelli L, Orizio G, Rossmann C, Ziegler L, Meyer L, et al. Crisis communication in the area of risk management: the CriCoRM project. Journal of Public Health Research 2013;2(20):118-21.
174. Schipp M. Australia's biosecurity procedures and preparedness. Microbiology Australia 2016;37(4):179-81.
175. Sealy T, Erickson B, Taboy C, Ströher U, Towner J, Andrews S, et al. Laboratory response to Ebola - West Africa and United States. 2016.
176. Selgelid M. Gain-of-function research: ethical analysis. Sci Eng Ethics 2016;22:923-64.
177. Sell T, Watson M. Federal agency biodefense funding, FY 2013-FY2014. Biosecurity and Bioterrorism: Biodefense Strategy, Practice, and Science 2013;11(3):196-216.
178. Serratosa J, Ribó O, Correia S, Pittman M. EFSA scientific risk assessment on animal health and welfare aspects of avian influenza. Avian Diseases 2004;51(1):501-3.
179. Shelby B, Cartagena D, McClee V, Gangadharan D, Weyant R. Transfer of select agents and toxins: 2003-2013. Health Security 2015;13(4):256-66.
180. Shelton S, Connor K, Uscher-Pines L, Pillemer F, Mullikin J, Kellermann A. Bioterrorism and biological threats dominate federal health security research; other priorities get scant attention. Health Affairs 2012;31(12):2755-63.
181. Shelton S, Nelson C, McLees A, Mumford K, Thomas C. Building performance-based accountability with limited empirical evidence: performance measurement for public health preparedness. Disaster Medicine and Public Health Preparedness 2013;373(4):373-9.
182. Shimazawa R, Ikeda M. Development of drug-approval regulations for medical countermeasures against CBRN agents in Japan. Health Security 2015;13(2):130-8.
183. Shreve C, Davis B, Fordham M. Integrating animal disease epidemics into disaster risk management. Disaster Prevention and Management 2016;25(4):506-19.
184. Shurtleff A, Garza N, Lackemeyer M, Carrion R, Griffiths A, Patterson J, et al. The impact of regulations, safety considerations and physical limitations on research progress at maximum biocontainment. Viruses 2012;4:3932-51.
185. Sijnesael P, Berg Lvd, Bleijs D, Odinot P, C dH, Jansen M, et al. Novel Dutch self-assessment Biosecurity Toolkit to identify biorisk gaps and to enhance biorisk awareness. Frontiers in Public Health 2014;2(197):1-5.
186. Sims L. Experience in control of avian influenza in Asia. Developments in Biologicals 2007;130:39-43.
187. Smith D, Novossiolova DMT. Microorganisms: good or evil, MIRRI provides biosecurity awareness. Curr Microbiol 2017;74:299-308.
188. Smolinski M, Crawley A, Olsen J. Finding outbreaks faster. Health Security 2017;15(2):215-20.
189. Stoto M. Measuring and assessing public health emergency preparedness. J Public Health Manag Pract 2013;19(5):16-21.
190. Stoto M. Biosurveillance capability requirements for the Global Health Security Agenda: lessons from the 2009 H1N1 pandemic. Biosecurity and Bioterrorism: Biodefense Strategy, Practice, and Science 2014;12(5):225-30.
191. Straetemans M, Buchholz U, Reiter S, Haas W, Krause G. Prioritization strategies for pandemic influenza vaccine in 27 countries of the European Union and the Global Health Security Action Group: a review. BMC Public Health 2007;7(236):1-12.
192. Su Y, Wu C, Lee T. Public health emergency response in Taiwan. Health Security 2017;15(2):137-43.
193. Sumpradit N, Chongtrakul P, Anuwong K, Pumtong S, Kongsomboon K, Butdeemee P, et al. Antibiotics smart use: a workable model for promoting the rational use of medicines in Thailand. Bulletin of the World Health Organization 2012;90:905-13.
194. Sun X, Keim M, Dong C, Mahany M, Guo X. A dynamic process of health risk assessment for business continuity management during the World Exposition Shanghai, China, 2010. Journal of Business Continuity & Emergency Planning 2014;7(4):347-64.
195. Sundqvist B, Bengtsson U, Wisselink H, Peeters B, van Rotterdam B, Kampert E, et al. Harmonization of European laboratory response networks by implementing CWA 15793: use of a gap analysis and an "insider" exercise as tools. Biosecurity and Bioterrorism: Biodefense Strategy, Practice, and Science 2013;11(1):36-44.
196. Swain K. The role of practical advice in bioterrorism news coverage. Health Security 2015;13(5):327-38.
197. Taboy C, Chapman W, Albetkova A, Kennedy S, Rayfield M. Integrated Disease Investigations and Surveillance planning: a systems approach to strengthening national surveillance and detection of events of public health importance in support of the International Health Regulations. BMC Public Health 2010;10(1):1-6.
198. Tegnell A, F VL, Baka A, Wallyn S, Hendriks J, Werner A, et al. Development of a matrix to evaluate the threat of biological agents used for bioterrorism. Cellular and Molecular Life Sciences 2006;63:2223-28.
199. Teng Y, Bi D, Xie G, Jin Y, Huang Y, Lin B, et al. Model-informed risk assessment for Zika virus outbreaks in the Asia-Paciﬁc regions. Journal of Infection 2017;74:484-91.
200. Thaler D, Cecchine G, Wong A, Jackson T. Building partner health capacity with US Military Forces. Washington, DC: RAND Corporation; 2012.
201. Thiberville S, Schilling S, De Iaco G, Fusco F, Thomson G, Maltezou H, et al. Diagnostic issues and capabilities in 48 isolation facilities in 16 European countries: data from EuroNHID surveys. BMC Research Notes 2012;5(1).
202. Tiangco M. Pro-poor HPAI risk reduction strategies: synthesis of country background papers. London, UK: Department for International Development; 2009.
203. Toner E, Nuzzo J, Shearer M, Watson C, Sell T, Cicero A. The Joint External Evaluation of Taiwan: the external evaluators' perspective. Health Security 2017;15(2):127-31.
204. Toner E, Waldhorn R, Franco C, Courtney B, Rambhia K, Norwood A, et al. Hospitals rising to the challenge: the first five years of the US Hospital Preparedness Program and priorities going forward. Baltimore, MD: Center for Biosecurity of UPMC; 2009.
205. Trevan T. Do not censor science in the name of biosecurity. Nature 2012;486:295.
206. U.S. Department of Health and Human Services. Possession, Use, and Transfer of Select Agents and Toxins; Biennial Review; Final Rule. Washington, DC. 2012.
207. Uhlenhaut C, Burger R, Schaade L. Protecting society. EMBO Reports 2013;14(1):25-30.
208. van Aken J. When risk outweighs benefit. EMBO Reports 2006;7:10-3.
209. Villarreal-Chávez C, Rivera-Cruz E. An update on avian influenza in Mexco. Avian Diseases 2003;47:1002-5.
210. Vogel K. Expert knowledge in intelligence assessments. International Security 2014;38(3):39-71.
211. Vong S, Samuel R, Gould P, El Sakka H, Rana B, Pinyowiwat V, et al. Assessment of Ebola virus disease preparedness in the WHO South-East Asia region. Bulletin of the World Health Organization 2016;94(12):913-24.
212. Walshe T, Burgman M. A framework for assessing and managing risks posed by emerging diseases. Risk Analysis 2010;30(2):236-49.
213. Wamala J, Okot C, Makumbi I, Natseri N, Kisakye A, Nanyunja M, et al. Assessment of core capacities for the International Health Regulations (IHR[2005]) – Uganda, 2009. BMC Public Health 2010;10.
214. Wang T, Hennessy D. Strategic interactions among private and public efforts when preventing and stamping out a highly infectious animal disease. Agricultural & Applied Economics Association Annual Meeting. Minneapolis, MN2014.
215. Waring S, Brown B. The threat of communicable diseases following natural disasters: a public health response. Disaster Management & Response 2005;3(2):41-7.
216. Warner K. Fighting pathophobia: how to construct constructive public engagement with biocontrol for nature without augmenting public fears. BioControl 2012;57(307-17).
217. Warren A. (Re)locating the border: Pre-entry tuberculosis (TB) screening of migrants to the UK. Geoforum 2013;48:156-64.
218. Warren A, Bell M, Budd L. Surveillance networks and spaces of governance: technological openness and international cooperation during the 2009 H1N1 pandemic. Annual Meeting of the Association of American Geographers. Washington, DC: Loughborough University; 2010.
219. Watkins R, Eagleson S, Hall R, Dailey L, Plant A. Approaches to the evaluation of outbreak detection methods. BMC Public Health 2006;2006(6).
220. Watson C, Watson M, Ackerman G, Gronvall G. Expert views on biological threat characterization for the US government: a Delphi study. Risk Analysis 2017;37(12):2389-404.
221. Wei X, Lin W, Hennessy D. Biosecurity and disease management in China’s animal agriculture sector. Food Policy 2015;54:52-64.
222. Wenzel J, Nusbaum K. Veterinary expertise in biosecurity and biological risk assessment. Vet Med Today: Disaster Medicine 2007;230(10):1476-80.
223. Westergaard J. Contingency planning: preparation of contingency plans. Zoonoses and Public Health 2007;55:42-9.
224. Wieland B, Dhollander S, Salman M, Koenen K. Qualitative risk assessment in a data-scarce environment: A model to assess the impact of control measures on spread of African Swine Fever. Preventive Veterinary Medicine 2011;99:4-14.
225. Wilinetz C. Implementing the new US dual-use policy. Science 2012;336(1525-7).
226. Wilson K, Brownstein J, Fidler D. Strengthening the International Health Regulations: lessons from the H1N1 pandemic. Health Policy and Planning 2010;25(505-9).
227. Wilson K, von Tigerstrom B, McDougall C. Protecting global health security through the International Health Regulations: requirements and challenges. CMAJ 2008;179(1):44-8.
228. World Health Organization. Responsible life sciences research for global health security: a guidance document. Geneva, Switzerland: World Health Organization; 2010.
229. World Health Organization. Quantitative risk assessment of the effects of climate change on selected causes of death, 2030s and 2050s. In: Hales S, Kovats S, Lloyd S, Campbell-Lendrum D, editors. Geneva, Switzerland: World Health Organization; 2014.
230. Xu Z, Zu Z, Zheng T, Zhang W, Xu Q, Liu J. Long-distance travel behaviours accelerate and aggravate the large-scale spatial spreading of infectious diseases. Computational and Mathematical Methods in Medicine 2014;2014:1-10.
231. Yamada S, Galat A. Typhoon Yolanda/Haiyan and climate justice. Disaster Medicine and Public Health Preparedness 2014;8(5):432-5.
232. Yang J, Teng H, Liu M, Li S. Taiwan's public health national laboratory system: success in influenza diagnosis and surveillance. Health Security 2017;15(2):154-64.
233. Yeh K, Adams M, Stamper P, Dasgupta D, Hewson R, Buck C, et al. National laboratory planning: developing sustainable biocontainment laboratories in limited resource areas. Health Security 2016;14(5):323-30.
234. Zaki A. Biosafety and biosecurity measures: management of biosafety level 3 facilities. International Journal of Antimicrobial Agents 2010;36:70-4.
235. Zaza S, Koonin L, Ajao A, Nystrom S, Branson R, Patel A, et al. A conceptual framework for allocation of federally stockpiled ventilators during large-scale public health emergencies. Health Security 2016;14(1):1-6.
236. Zhen X, Pavlin B, Squires R, Chinnayah T, Konings F, Lee C, et al. Ebola preparedness in the Western Pacific Region, 2014. Western Pacific Surveillance and Response Journal 2015;6(1):66-72.
237. Zuckerman M. Biosecurity: a 21st century challenge. New York City, NY: Carnegie Corporation of New York; 2005.
